# Perinatal risk factors for hepatoblastoma

**DOI:** 10.1038/sj.bjc.6604335

**Published:** 2008-04-08

**Authors:** L G Spector, K J Johnson, J T Soler, S E Puumala

**Affiliations:** 1 Division of Epidemiology/Clinical Research, Department of Pediatrics, University of Minnesota, 420 Delaware Street, SE MMC 715, Minneapolis, MN 55455, USA; 2 Cancer Center, University of Minnesota, 420 Delaware Street, SE MMC 715, Minneapolis, MN 55455, USA; 3 Minnesota Department of Health, PO Box 64975, St Paul, MN 55164-0975, USA

**Keywords:** paediatrics, case–control studies, medical record linkage, prematurity

## Abstract

We confirmed the strong association of hepatoblastoma with very low birth weight (relative risk <1000 g *vs* ⩾2000 g=25.6; 95% confidence interval: 7.70–85.0) and demonstrated independent associations with congenital abnormalities and maternal Asian race in a population-based Minnesota study that included 36 cases and 7788 controls.

Hepatoblastoma is a rare embryonal tumour that comprises most cases of liver cancer in children aged 0–5 years in the United States (Ries *et al*, 1999). Although its causes are mostly obscure, it is apparent that very low birth weight (VLBW), generally defined as <1500 g, is a potent risk factor (Tanimura *et al*, 1998; Reynolds *et al*, 2004; Ansell *et al*, 2005; McLaughlin *et al*, 2006). Hepatoblastoma incidence doubled between 1975 and 1999 (Spector *et al*, 2004), possibly related to the concomitant rise in prevalence of VLBW infants and a marked drop in their mortality (CDC, 2002). These observations may indicate that some component of treatment for prematurity is carcinogenic or, alternatively, that the aetiology may overlap with that of VLBW. To expand a sparse literature, we examined the relation between hepatoblastoma and birth characteristics in Minnesota.

## MATERIALS AND METHODS

The methods used in this case-cohort study have previously been described (Puumala *et al*, 2008). Briefly, we matched Minnesota Cancer Surveillance System (MCSS) records of incident first cancers diagnosed in children aged 28 days to 14 years during 1988–2004 to birth records using probabilistic record linkage (Jaro, 1995). For each of the 2188 successfully linked cases (out of 2655 total) we randomly selected four birth records of children born in the same year and who survived at least 28 days past birth; this comparison group of 8752 subjects is referred to as the subcohort. In this analysis, matching cases were compared to all subcohort members born in 1982 (i.e., the earliest year a case would have been born) or later to improve study power.

Exposure variables derived from Minnesota birth records are shown in Table 1. Some variables were not available during the entire study period. Birth weight was divided into categories of <1000, 1000–1999, and ⩾2000 g to maximize the number of cases in each stratum. Race was classified as white or non-white. Other variables were categorized using customary cut offs (Ries *et al*, 1999).

We calculated hazard ratios (HRs) and 95% confidence intervals (CIs) using stratified Cox regression models using SAS 9.1 (SAS institute Inc., Cary, NC, USA) (Langholz and Jiao, 2007). The number of cases precluded a full multivariate model. However, birth weight is a known risk factor; all variables were modelled adjusting for birth weight as well as for year of birth and sex.

## RESULTS

Of 39 cases of hepatoblastoma identified by MCSS, 36 (92.3%) linked to birth records. Cases were compared to 7788 members of the subcohort. Exposure frequencies and adjusted HRs are presented in Table 1; where cell size was <4, we reported frequencies but did not calculate HRs. There were strong associations with birth weight <1000 g (HR=25.6; 95% CI: 7.70–85.0) and 1000–1999 g (HR=9.15; 95% CI: 3.09–27.1) compared to ⩾2000 g, their magnitude with each category of low birth weight was lessened, but remained highly significant, when adjusting for covariates (data not shown). Significant univariate associations were noted with maternal and paternal Hispanic ethnicity, maternal birthplace outside the US, intrauterine procedures during pregnancy, male sex, gestational age <37 weeks, 1 and 5 min apgar scores, assisted ventilation, and congenital abnormalities (data not shown). However, only associations with congenital abnormalities (HR=5.87; 95% CI: 1.88–18.3), paternal Hispanic ethnicity (HR=4.18; 95% CI: 1.22–14.3), and maternal birthplace outside the US (HR=3.55; 95% CI: 1.51–8.32) remained significant after adjustment. The association of foreign maternal birthplace reflected the disproportionate number of cases with mothers from Southeast Asia. Accordingly, there was a significant adjusted association of maternal Asian race with hepatoblastoma (HR=3.86; 95% CI: 1.30–11.52).

## DISCUSSION

**Figure 1:**
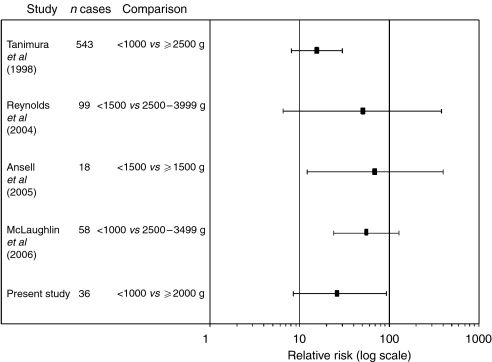
Relative risk of hepatoblastoma comparing very low to moderate birth weight children in five studies.

Very low birth weight has emerged over the past 15 years as a potent risk factor for hepatoblastoma (Feusner *et al*, 1998; Tanimura *et al*, 1998; Reynolds *et al*, 2004; Ansell *et al*, 2005; McLaughlin *et al*, 2006). We have now confirmed this association in the paediatric population of Minnesota. Unadjusted relative risks between 16 and 70 comparing very low to moderate birth weight children have been reported in studies from the United Kingdom, Japan, and now three US states (Figure 1). This striking association thus appears across industrialized nations.

Rather than being causative *per se*, VLBW likely signals the involvement of correlated factors. Limited multivariate analyses in this study and others (Reynolds *et al*, 2004; McLaughlin *et al*, 2006) have begun to tease apart the role of other characteristics from that of VLBW. Notably, preterm birth (<37 weeks) was not an independent risk factor in any of the studies after adjustment.

We found a strong association with congenital abnormalities, which was attenuated but still present when controlling for VLBW. One specific abnormality, an omphalocele, was recorded, which may be indicative of Beckwith–Wiedemann syndrome, an overgrowth disorder which is known to increase the risk of hepatoblastoma (Ranzini *et al*, 1997; DeBaun and Tucker, 1998). The remaining five abnormalities were nonspecific (one central nervous system, one urogenital, and three ‘others’). Higher than expected proportions of congenital abnormalities have previously been noted among cases (Narod *et al*, 1997; Ansell *et al*, 2005), but without factoring in birth weight.

The association of maternal Asian race, specifically of Southeast Asian ancestry, with hepatoblastoma was unexpected. Similar findings were not noted in studies in California (Reynolds *et al*, 2004) and New York (McLaughlin *et al*, 2006), and there was not an elevated rate in Ho Chi Minh City, Vietnam (Nguyen *et al*, 2000). Our observation was therefore novel and independent of birth weight. We also observed an independent association of paternal, but not maternal, Hispanic ethnicity. This finding may have been an artifact, as its significance was dependent on two cases for which paternal ethnicity was missing.

Maternal hypertension (Roberts *et al*, 2003), maternal tobacco use (Horta *et al*, 1997), and conception by assisted reproductive technology (Schieve *et al*, 2002) are known to reduce birth weight and have been examined in other studies. An excess of maternal pre-eclampsia, without adjustment for VLBW, has been noted (Ansell *et al*, 2005). Three previous studies have found that maternal smoking significantly raised offspring risk (Pang *et al*, 2003; Sorahan and Lancashire, 2004; McLaughlin *et al*, 2006), whereas a fourth did not (Buckley *et al*, 1989); associations remained after adjustment for VLBW in two studies (Pang *et al*, 2003; Spector and Ross, 2003; McLaughlin *et al*, 2006). Lastly, a ninefold increased risk of hepatoblastoma was reported among children born following infertility treatment, which was independent of birth weight (McLaughlin *et al*, 2006). That maternal smoking and conception by assisted reproductive technology remain as risk factors after adjustment for VLBW suggests that if causal relationships exist, they operate independently of birth weight.

Use of population-based registry data was the major strength of this study, as any misclassification would most likely be non-differential and HRs would be underestimated. Birth characteristics and delivery methods are reliably recorded in birth records whereas other factors, including congenital abnormalities, are substantially underreported (Northam and Knapp, 2006), contributing to sparse data for several variables. Although cases that occurred among outmigrating children or those who resided in Minnesota during 1982–1987 would have been missed, this occurrence is unlikely given the very low incidence of hepatoblastoma. Lastly, the small number of cases resulted in a reduced ability to perform multivariate analyses.

The strong association of hepatoblastoma with VLBW has been robust to adjustment for other factors. That control for several prenatal determinants of small infant size has not explained the VLBW association may indicate that postnatal treatment is the causative correlate. Although three very small case–control studies of neonatal medical history among VLBW infants preliminarily suggest greater oxygen exposure in cases (Maruyama *et al*, 1999, 2000; Oue *et al*, 2003), larger studies are plainly required. Therefore, a multicenter case–control study has been initiated (National Institutes of Health Grant R01CA111355; L Spector, Principal Investigator) that will examine risk factors for hepatoblastoma, with a special focus on VLBW infants.

## Figures and Tables

**Table 1:** Associations of demographic, pregnancy, and birth characteristics with hepatoblastoma adjusted for birth weight, birth year, and sex — Minnesota, 1982–2004

	**Subcohort (*n*=7788)**	**Cases (*n*=36)**		
**Characteristic**	***N* (%)**	***N* (%)**	**HR^a^**	**95% CI**
*Birth characteristics*				
Birth weight (g)				
>2000	7606 (98.0)	28 (77.8)	1	
1000–1999	124 (1.6)	4 (11.1)	9.15	3.09–27.1
<1000	31 (0.4)	4 (11.1)	25.6	7.70–85.0
Sex				
Female	3778 (48.5)	11 (30.6)	1	
Male	4010 (51.5)	25 (69.4)	2.03	0.98–4.19
Gestational age (weeks)^b^				
⩾37	6888 (91.6)	25 (69.4)	1	
<37	631 (8.4)	11 (30.6)	1.70	0.56–5.13
Size for gestational age				
Small	252 (3.4)	0 (0.0)	—	
Average	5427 (72.4)	29 (80.6)	—	
Large	1821 (24.3)	7 (19.4)	—	
Multiple birth				
No	7592 (97.5)	34 (94.4)	—	
Yes	196 (2.5)	2 (5.6)	—	
Apgar score (1 min)				
>7	5725 (76.6)	19 (52.8)	1	
⩽7	1746 (23.4)	17 (47.2)	1.99	0.95–4.16
Apgar score (5 min)				
>7	7192 (96.4)	29 (80.6)	1	
<7	265 (3.6)	7 (19.4)	2.24	0.70–7.11
Assisted ventilation^c^				
No	4549 (98.3)	25 (86.2)	1	
Yes	79 (1.7)	4 (13.8)	2.00	0.49–8.19
Congenital abnormality				
No	7676 (98.6)	31 (86.1)	1	
Yes	112 (1.4)	5 (13.9)	5.87	1.88–18.3
				
*Index pregnancy history*				
Adequacy of prenatal care				
Adequate	4530 (67.9)	24 (75.0)	1	
Intermediate	1488 (22.3)	4 (12.5)	0.61	0.21–1.78
Inadequate	658 (9.9)	4 (12.5)	1.11	0.36–3.43
Weight gain (pounds)^c^				
⩽24	1029 (26.7)	7 (25.9)	1	
25–30	1295 (33.6)	10 (37.0)	1.36	0.48–3.83
>30	1526 (39.6)	10 (37.0)	1.40	0.48–4.07
Intrauterine procedures				
No	7271 (97.4)	32 (88.9)	1	
Yes	193 (2.6)	4 (11.1)	2.39	0.68–8.41
Induction of labor^c^				
No	3439 (71.6)	20 (66.7)	1	
Yes	1361 (28.4)	10 (33.3)	1.32	0.60–2.93
Type of delivery				
Vaginal	6162 (82.0)	26 (72.2)	1	
Caesarean section	1355 (18.0)	10 (27.8)	1.26	0.58–2.77
				
*Maternal reproductive history*				
Prior live births				
0	3062 (39.9)	16 (44.4)	1	
1–2	3799 (49.5)	11 (30.6)	0.62	0.28–1.35
⩾3	808 (10.5)	9 (25.0)	2.07	0.87–4.92
Interval since last live birth (years)				
No prior birth	3062 (40.1)	16 (44.4)	—	
⩽3	2966 (38.9)	18 (50.0)	—	
>3	1600 (21.0)	2 (5.6)	—	
Prior fetal losses				
None	5919 (77.5)	30 (83.3)	1	
Any	1717 (22.5)	6 (16.7)	0.60	0.24–1.49
				
*Parental demographics*				
Mother's age (years)				
<25	2358 (30.3)	11 (30.6)	1.27	0.53–3.08
25–29	2708 (34.8)	10 (27.8)	1	
⩾30	2722 (35.0)	15 (41.7)	1.31	0.57–3.01
Father's age (years)				
<30	3347 (47.5)	11 (33.3)	1	
⩾30	3693 (52.5)	22 (66.7)	1.55	0.74–3.25
Mother's race				
White	7010 (90.9)	30 (83.3)	1	
Non-white	700 (9.1)	6 (16.7)	1.56	0.63–3.89
Mother's ethnicity^c^				
Non-Hispanic	4475 (96.5)	26 (86.7)	1	
Hispanic	160 (3.5)	4 (13.3)	2.96	0.88–10.0
Mother's birthplace				
United States	7297 (93.8)	28 (77.8)	1	
Elsewhere	485 (6.2)	8 (22.2)	3.55	1.51–8.32
Father's race				
White	6381 (92.5)	28 (87.5)	1	
Nonwhite	521 (7.5)	4 (12.5)	1.44	0.49–4.29
Father's ethnicity^c^				
Non-Hispanic	3996 (97.0)	24 (85.7)	1	
Hispanic	125 (3.0)	4 (14.3)	4.18	1.22–14.3
Mother's education (years)				
<12	782 (10.6)	8 (22.9)	1.60	0.64–4.02
12	2706 (36.8)	14 (40.0)	1	
>12	3867 (52.6)	13 (37.1)	0.61	0.28–1.32
Father's education (years)				
⩽12	2840 (44.2)	13 (43.3)	1	
>12	3587 (55.8)	17 (56.7)	1.03	0.49–2.16

Abbreviations: CI=confidence interval; HR= Hazard ratios; LNMP=last normal menstrual period

a Hazard ratios adjusted for birth weight, birth year, and sex.

b Imputed gestational age based on LNMP when available and physician's estimate when LNMP was missing.

c Collected in birth years 1989–2004.
